# TbFRP, a novel FYVE-domain containing phosphoinositide-binding Ras-like GTPase from trypanosomes

**DOI:** 10.1016/j.exppara.2012.11.007

**Published:** 2013-03

**Authors:** Vincent O. Adung’a, Mark C. Field

**Affiliations:** Department of Pathology, University of Cambridge, Tennis Court Road, Cambridge CB2 1QP, UK

**Keywords:** Endocytosis, Trypanosoma, Phosphoinositide, Intracellular transport, Proteolysis, GTPase

## Abstract

Ras-like small GTPases are regulatory proteins that control multiple aspects of cellular function, and are particularly prevalent in vesicular transport. A proportion of GTPase paralogs appear restricted to certain eukaryote lineages, suggesting roles specific to a restricted lineage, and hence potentially reflecting adaptation to individual lifestyles or ecological niche. Here we describe the role of a GTPase, TbFRP, a FYVE domain N-terminally fused to a Ras-like GTPase, originally identified in *Trypanosoma brucei*. As FYVE-domains specifically bind phosphoinositol 3-phosphate (PI3P), which associates with endosomes, we suggest that TbFRP may unite phosphoinositide and small G protein endosomal signaling in trypanosomatids. TbFRP orthologs are present throughout the Euglenazoa suggesting that FRP has functions throughout the group. We show that the FYVE domain of TbFRP is functional in PI3P-dependent membrane targeting and localizes at the endosomal region. Further, while TbFRP is apparently non-essential, knockdown and immunochemical evidence indicates that TbFRP is rapidly cleaved upon synthesis, releasing the GTPase and FYVE-domains. Finally, TbFRP expression at both mRNA and protein levels is cell density-dependent. Together, these data suggest that TbFRP is an endocytic GTPase with a highly unusual mechanism of action that involves proteolysis of the nascent protein and membrane targeting via PI3P.

## Graphical abstract

**Graphical abstract:**

**Figure:**
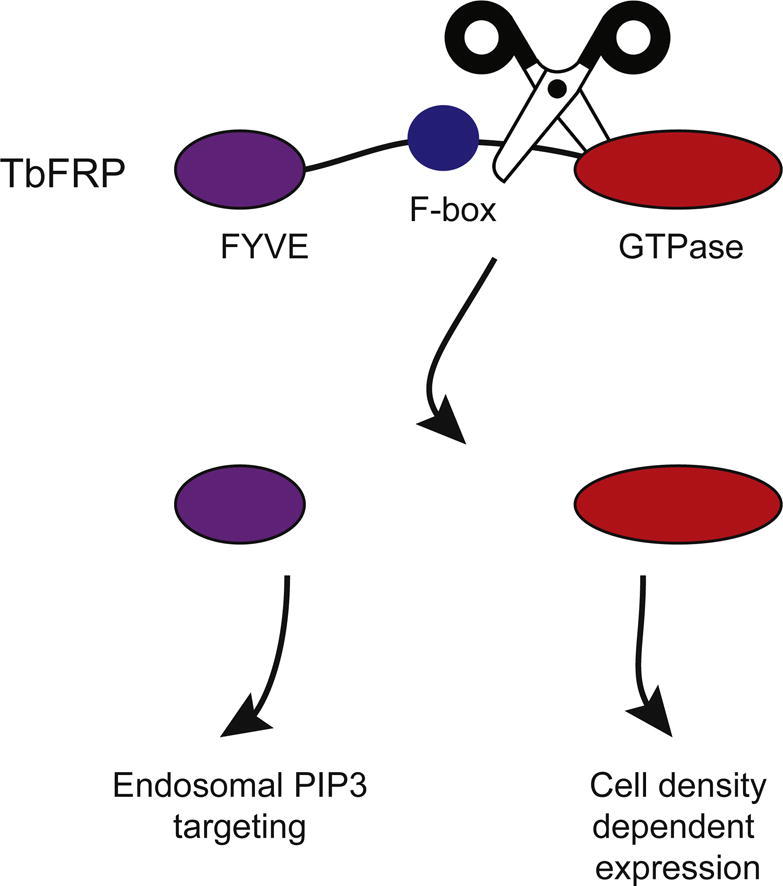

**Highlights:**

► TbFRP is a GTPase with a novel architecture (FYVE-GTPase). ► Orthologs of TbFRP are restricted to Euglenozoa. ► TbFRP is rapidly cleaved to release the GTPase domain. ► The TbFRP FYVE domain is functional in PIP3-binding. ► Expression levels of TbFRP are controlled in a cell density dependent manner.

## 1 Introduction

Signal transduction in eukaryotes is mediated by multiple mechanisms; amongst the most important are pathways mediated by small Ras-like GTPases and phosphoinositides (Schimmöller et al., 1998; Zerial and McBride, 2001; Haucke, 2005). Both mechanisms interface with kinase cascades and in higher eukaryotes at least lead to activation of specific gene cohorts, associated with differentiation and/or proliferative programs. However, small GTPases have a particularly prominent role in intracellular transport, with the Rab, ARF and Ran families all participating directly in the control of macromolecular targeting (Zerial and McBride, 2001; Grosshans et al., 2006; Mizuno-Yamasaki et al., 2012). Rab GTPases are master regulators of intracellular membrane transport and are involved in vesicle formation, migration, docking and fusion, making them key players in trafficking between the organelles of the endocytic and secretory pathways (Zerial and McBride, 2001). Further, phosphoinositide subspecies are associated with specific endomembrane compartments. For example, phosphoinositol 3-phosphate (PI3P) is present at endosomal membranes of Metazoa, fungi, plants and trypanosomes (Haucke, 2005; Hall et al., 2006), with phosphoinositide 4,5 bisphosphate being associated with the plasma membrane (Ford et al., 2002; Aguilar et al., 2006). These lipid subspecies are present at low concentrations in the membrane bilayer, but serve to facilitate the binding of proteins bearing specific domains recognizing these phosphoinositide phosphate (PIP) subclasses.

Interactions between Rab and PI-mediated signaling are complex, and remain incompletely understood. PIP versatility is reflected in the spectrum of binding modules recognizing these subspecies, and which include ENTH and ANTH domains, FYVE domains, pleckstrin homology (PH) domains, phox (PX) domains and others. The polypeptides containing these lipid-binding domains are mostly multi-domain proteins, and hence can synergistically recruit additional proteins to their sites of interaction. One of the best characterized Rab/PIP interactions concerns Rab5. Rab5 is recruited to early endosome membranes by early endosome antigen 1 (EEA1), a coiled coil domain protein that itself is targeted to PI3P via its FYVE domain (Simonsen et al., 1998). EEA1 also recruits Rab22 (Kauppi et al., 2002), syntaxin 6 (Simonsen et al., 1999) and syntaxin 13 (McBride et al., 1999; Mills et al., 2001), allowing the formation of protein complexes that drive subsequent events. Vps34, an evolutionarily conserved vacuolar phosphoinositide 3-kinase, generates PI3P and is itself recruited to membranes by Rab5.

In Trypanosoma brucei, the causative agent of African sleeping sickness, the absence of promoter driven mechanisms for control of gene expression also suggests unusual signal transduction mechanisms may be present (Field, 2005). A complex repertoire of protein and phosphoinositol kinases has been described (Parsons et al., 2005; Hall et al., 2006), but a detailed understanding of the pathways that these molecules populate is lacking. Further also, T. brucei possesses a complex repertoire of small GTPases (Field, 2005; Field and O’Reilly, 2008), but again many lack an obvious function based on in silico analysis. The majority of the Rab GTPases are characterized in some detail (Field et al., 1998; Jeffries et al., 2001, 2002; Pal et al., 2002; Hall et al., 2005; Lumb et al., 2011), while several ARFs and a Rho-like protein are partially characterized (Price et al., 2007; Abbasi et al., 2011). The Vps34 ortholog is required for synthesis of endosomal membrane PI3P in trypanosomes and Rab5 for endosomal trafficking (Hall et al., 2006), but a trypanosomal EEA1 ortholog is absent, indicating that the mode of tethering/docking and fusion of membrane-derived endocytic vesicles is likely novel.

We identified an open reading frame encoding a unique Ras-like GTPase in the trypanosome genome, which possesses an N-terminal FYVE-domain and F-box, which we designated TbFRP (Field, 2005): TbFRP may provide an alternate mechanism for endosomal tethering, with the potential to integrate small GTPase and PIP signaling pathways in trypanosomes. Here we show that TbFRP orthologs are restricted to Euglenozoa. Using gene silencing and genomic tagging we demonstrate that TbFRP is non-essential but that the FYVE-domain is functional in PI3P recognition, suggesting endosomal targeting. Furthermore, we also find that the TbFRP protein is cleaved, with little evidence that the FYVE and GTPase domains remain associated to a significant degree. Most significantly, TbFRP expression is density and/or media condition dependent, suggesting a potential role in conditioning of the trypanosome endosomal system in a differentiation-dependent manner, with potentially important functions for environmental sensing by other kinetoplastids and Euglenids.

## 2 Materials and methods

### 2.1 Informatics

Sequence data for trypanosomatids were retrieved from tritrypdb (http://tritrypdb.org/tritrypdb/). Additional data for Bodo saltans were from genedb (http://www.genedb.org/Homepage/Bsaltans), for Phytomonas serpens data was donated by Dr. L. Koreny (University of Cambridge). Data for Euglena gracilis and Trypanosoma grayi were from in house datasets obtained by 454 and Illumina sequencing respectively (MCF, unpublished). Alignments were performed in Muscle (http://www.ebi.ac.uk/Tools/msa/muscle/) (Edgar, 2004), and edited with Mesquite (http://mesquiteproject.org/mesquite/mesquite.html) (Maddison and Maddison, 2011) to remove highly divergent regions. Phylogenetic reconstruction was performed with MrBayes (Huelsenbeck and Ronquist, 2001), PhyML (Guindon et al., 2010) and RaxML (Silvestro and Michalak, 2011) as described (Elias et al., 2012).

### 2.2 Production of TbFRP polyclonal antibodies

TbFRP rabbit antiserum was generated against purified recombinant TbFRP GTPase domain. The recombinant protein was expressed in BL21 Escherichia coli using the pGEX-3T expression vector system (GE Healthcare) and purified on glutathione-sepharose according to the manufacturer’s protocol. Affinity purification and characterization of the antibodies was exactly as described previously (Abbasi et al., 2011).

### 2.3 Cell culture

The bloodstream form trypanosome (BSF) line T. brucei Lister 427 were cultured in HMI 9 at 37 °C, 5% CO_2_ while procyclic forms (PCF) T. brucei 427 in SDM 79 media at 26 °C, both media supplemented with 10% tetracycline free-fetal bovine serum (Autogen Bioclear). BSF lines expressing HA tagged recombinant protein were continuously cultured in presence of 2.5 μg ml^−1^ neomycin (Sigma). For RNAi analysis, single marker T7 RNAP/TETR BSF cells (SMB; Wirtz et al., 1999) were used. SMB cells were continuously maintained in the presence of 2.5 μg ml^−1^ neomycin and neomycin plus hygromycin (Sigma; 2.5 μg ml^−1^) for the RNAi lines. The density of RNAi lines was maintained between 1 × 10^5^ and 2 × 10^6^ cells ml^−1^. To determine the effects of TbFRP ablation by RNAi, proliferation curves, RNA and protein levels were compared between cultures with and without the addition of tetracycline (1 μg ml^−1^).

### 2.4 Quantitative real time (qRT)-PCR and Western blot

qRT-PCR was performed on equal amount of cDNA according to Koumandou et al. (2008). The primer sequences used are given in Table S1. All analyses were performed in triplicate with at least two biological replicates. For Western blotting, cells were washed in phosphate-buffered saline (PBS), and resuspended in 1× SDS sample buffer prewarmed to 95 °C and boiled for 10 min. Lysates were resolved on 12% SDS–PAGE gels and proteins transferred onto Hybond-ECL nitrocellulose membranes (Amersham Biosciences) overnight by wet transfer at 12 V (Abbasi et al., 2011). The membranes were blocked and processed following standard procedures (Abbasi et al., 2011). Polyclonal anti-TbFRP antibody was used at 1:5000, rabbit anti-BIP (kind gift of James D. Bangs) at 1:10,000, murine monoclonal anti-HA, rabbit anti-mouse immunoglobulin and goat anti-rabbit horseradish peroxidase secondary antibodies (all Sigma) were used at 1:10,000.

### 2.5 Plasmid construction and transfection

For ectopic expression of C-terminal HA-tagged TbFRP in the pXS519 vector (Pal et al., 2002), the TbFRP ORF was amplified using the primers TbFRP For and TbFRP Rev (Table S1), and the product digested with HindIII and ApaI and ligated into the pXS519 vector linearized with the same enzymes. Similarly, the TbFRP FYVE domain alone and the TbFRP FYVE plus F-box domains were amplified using a common forward primer (TbFRP For) and their respective reverse primers, TbFRP-FYVE Rev and TbFRP-FYVE-F-box Rev, and subsequently ligated into the pXS519 vector. 10 μg of XhoI linearized plasmid were used for transfections. For genomic HA-tagging of the TbFRP ORF the method by Oberhozer (Oberhozer et al., 2006) was applied. Briefly, a tagging construct was amplified from pMOTag3H using TbFRP Tag For and Rev and the products precipitated with ethanol. To transfect BSF 427, ∼15 μg of DNA resuspended in sterile TE buffer was used and transformants selected with neomycin as described (Abbasi et al., 2011).

### 2.6 Immunofluorescence

Cells were harvested at 800*g* for 10 min at 4 °C, washed with ice-cold Voorheis‘s-modified PBS (vPBS) and stained according to Field et al. (2004) prior to mounting with Vectashield medium supplemented with 4′,6-diamidino-2-phenylindole (DAPI) (Vector Laboratories, Inc). The primary antibodies were diluted in blocking solution and used at the following concentrations: mouse and rabbit anti-HA at 1:1000 (Invitrogen), rabbit anti-clathrin at 1:250, anti-mouse Oregon Green or Red and anti-rabbit Oregon Green or Red at 1:1000 (all from Molecular Probes). Visualization was done on a Nikon Eclipse E600 epifluorescence microscope with a Hamamatsu ORCA CCD camera and images captured using Metamorph software (Universal Imaging Corp.). Final processing for presentation was done using Adobe Photoshop 13.0 (Adobe Systems Inc.).

### 2.7 Subcellular fractionation

Cells (1 × 10^8^) were washed twice in ice-cold PBS, resuspended in 100 μl of hypotonic lysis buffer (10 mM Tris–HCl, pH 7.5), incubated for 5 min on ice and centrifuged at 20,000*g* for 10 min at 4 °C. The supernatant was transferred to a fresh tube, and an equal volume of 2× SDS sample buffer added before incubation at 95 °C for 10 min. The pellet was washed in 100 μl of hypotonic lysis buffer and resuspended in 100 μl of ice-cold sample lysis buffer (50 mM Tris–HCl, pH 7.7; 150 mM NaCl and 1% Nonidet P-40) followed by 25 min incubation in ice. An equal volume of 2× SDS sample buffer was added and incubation at 95 °C for 10 min. Both fractions were resolved on SDS–PAGE.

### 2.8 Pharmacological treatments

Cells were treated with wortmannin as previously described (Hall et al., 2006) and the GFP-2xFYVE cell line used as a positive control. Cells expressing GFP-2xFYVE and HA-tagged TbFRP-FYVE and TbFRP-FYVE-F-box were cultured in the presence of wortmannin (Sigma) at 3 μM for one to two hours. The localization of the tagged proteins was determined by immunofluorescence as above.

### 2.9 Cell density and TbFRP expression level

To determine the levels of TbFRP during proliferation, BSF (1 × 10^5^ ml^−1^) and PCF (2 × 10^6^ ml^−1^) trypanosomes were cultured in HMI-9 and SDM79 medium respectively for 72 h without passage. Samples were withdrawn after every 24 h and levels of TbFRP determined using Image J software after Western blotting. At same time points, TbFRP mRNA levels were determined by qRT-PCR. To determine the effect of medium composition on TbFRP levels, trypanosomes at 72 h (as above) were harvested, resuspended in fresh media, and cultured for 24 h; TbFRP levels were determined at 0, 6, 12 and 24 h by Western blotting. In addition, the cells at 24 h time point were harvested, washed appropriately and cultured in spent medium from 72 h cultures or fresh medium. Subsequently, the level of TbFRP was monitored at 6, 12 and 24 h by Western blotting.

## 3 Results

### 3.1 A GTPase with unusual architecture present in trypanosomes and euglenids

**Fig. 1:**
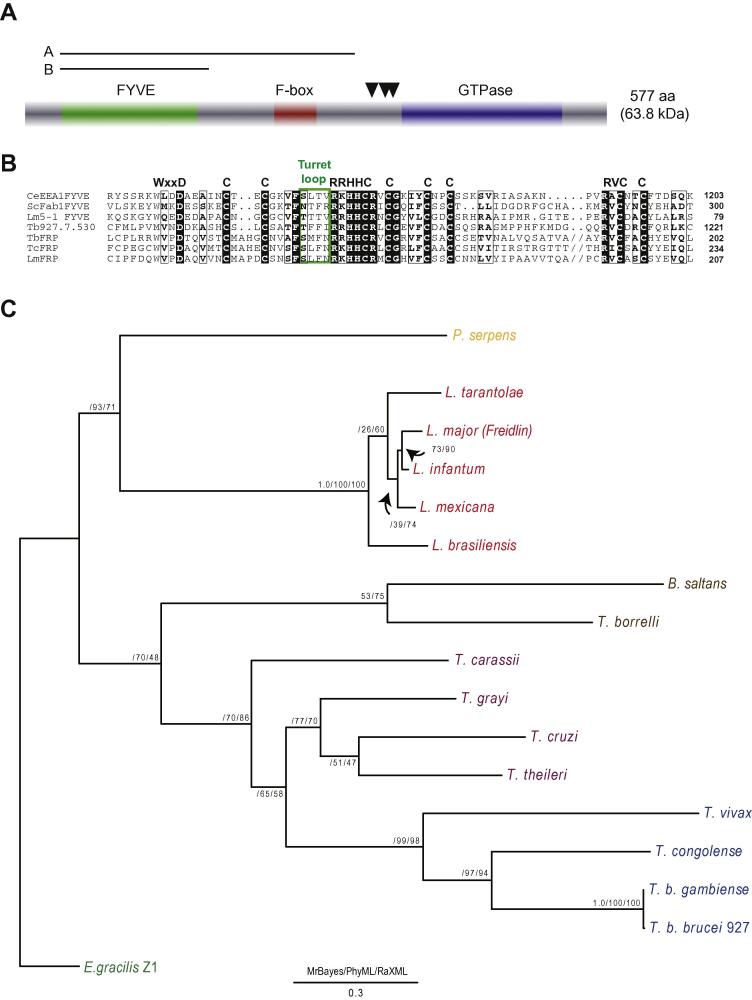
TbFRP domain organization and representation in trypanosomatids and Euglenids. Panel A. Trypanosoma brucei TbFRP has FYVE, F-box and GTPase domains at the N, central and C-terminus, respectively. This organization is unique to trypanosomatids and related Euglenids. Lines labeled A and B are the regions corresponding to the sequences targeted by RNAi. Arrowheads designate potential proteolytic cleavage sites. Panel B. FYVE domain sequence alignment showing conserved WxxD, R(R/K)HHCR, RVC motifs and cysteine residues. The turret loop (green) represents four amino acids before the R(R/K)HHCR. TbFRP FYVE has an insertion (−//−) since there are at least 68 amino acid residues between the cysteine residue at RrHHCR motif + 10 and the RVC motifs in trypanosomatid FRP as compared to 13 to 23 residues in other FYVE domains. Tb (T. brucei), Tc (T. cruzi), Lm (L. major), CeEEA1 (Caenorhabditis elegans Early Endosome Antigen 1), ScFab1 (Sacchromyces cerevisiae Fab1-formation of aploid and binucleate cells). For LmFYVE5–1, see Mertens et al. (2007). Tb927.7.530 is the gene ID of a FYVE domain containing T. brucei protein. Panel C: phylogenetic reconstruction of FRP evolutionary history from 17 euglenid genomes. Organisms are color coded with photosynthetic organisms in green, African trypanosome clade in blue, South American-related clade in purple, Phytomonads in yellow, Leishmania in red and a putative B. saltans clade in brown. Statistical support values are shown for Mr. Bayes, PhyML and RaXML reconstructions; the PhyML topology is shown. Note that the extreme long branch of E. gracilis is likely exaggerated due to incomplete sequence.

TbFRP (Tb927.7.3790) has a predicted molecular weight of 63.8 kDa and contains FYVE and GTPase domains at the N- and C-termini, respectively and a central F-box domain (Fig. 1A) (Field, 2005). This domain organization and composition is unique to trypanosomatids and related euglenids (Field, 2005; Field and O’Reilly, 2008). Additional searches confirmed exclusivity of FRP orthologs to kinetoplastida and extended the phylogenetic depth to include B. saltans and E. gracilis, a basal euglenid (Fig. 1), hence suggesting an origin for FRP prior to speciation of the Euglenozoa. By contrast, searches of the Naegleria gruberi genome failed to yield a potential ortholog. Therefore FRP is an ancient feature of the Euglenozoa and likely arose during the segregation of Euglenids from other excavates. The GTPase domain alone is most closely related to TbRab18 in trypanosomes based on reverse BLAST analysis (data not shown), which is located at the Golgi complex and may function in retrograde transport (Jeffries et al., 2002), and Ypt6 in Saccharomyces cerevisiae, which also participates in retrograde endosome to Golgi trafficking (Luo and Gallwitz, 2003). These data suggest that FRP may have arisen by the fusion of an ancestral Rab18/Ypt6 with a FYVE-domain containing ORF in an early Euglenid, but the origin of the FYVE portion is unclear.

Most Ras-like GTPases are targeted to membranes through a combination of prenylation and palmitoylation at the C-terminus via processing of a CAAX motif (Hancock et al., 1989). However, in TbFRP, the C-terminal amino acid residues are VLLD, and hence prenylation is unlikely. Further, a CAAX motif is absent from either within the predicted ORF or potentially encoded within the predicted 3′ UTR, suggesting that the absence of a CAAX motif is not due to misannotation, and opening up the possibility that the N-terminal FYVE domain mediates membrane association. The FYVE domain is cysteine-rich and mediates high affinity specific binding to PI3P, a PI highly enriched in early endosomes (Gaullier et al., 2000; Gillooly et al., 2000; Hayakawa et al., 2007), multivesicular bodies (MVB) and the Golgi complex (Gillooly et al., 2000).

FYVE domain motifs, namely WxxD, R(R/K)HHCR, RVC and eight cysteine residues, are essential for interaction with PI3P and coordination of two zinc ions (Stenmark et al., 1996; Gillooly et al., 2001; Lee et al., 2006). These motifs and cysteine residues are fully conserved in FRP (Fig. 1B). However, between the conserved cysteine, at R(R/K)HHCR + 10 residues and the RVC motif, there is an insertion of between 68 and 127 amino acid residues in Trypanosoma and Leishmania species respectively, compared to 13–23 residues in other well characterized FYVE domains (Fig. 1B, S1, S2A and Table S2). A similar insertion was observed in FYVE domains of three other T. brucei proteins encoded by Tb11.61.0730, Tb11.47.0002 and Tb927.7.690 and their orthologs in additional trypanosomatids (see Table S2), hence this insertion is not FRP specific. The FYVE insertions vary between gene and organism, with Leishmania exhibiting slightly larger insertions than trypanosomes, a common feature in these parasites (Myler, 2008). In addition, a specific variant FYVE domain that has an RxxC motif (xx represents [L/H/R/P]/[V/A]) as opposed to an RVC motif and no insertion (Fig. S2B) was also observed in Tb11.01.6980 and its orthologs.

A FYVE-domain insertion, similar to TbFRP, was observed in representative proteins of all eukaryotic supergroups except Plantae. Notably, this large insert is present in Rabenosyn-5 and Phytophthora infestans phosphatidylinositol kinase (PIK-D) (Table S2; Fig. S2A). The T. brucei ortholog of P. infestans PIK-D (Tb11.01.6980) however, lacks the insertion, an indication of apparent plasticity. Insects, more specifically several Drosophila species, Aedes and Anopheles, have a FYVE domain with an insertion N-terminal to the ‘turret loop’ (Fig. S2C), a region implicated in electrostatic interaction with the membrane (Dumas et al., 2001). Further, the Pichia stipitis Bright DNA-binding protein (trA3GH95) has a FYVE domain with insertions in both positions represented above (data not shown). Therefore, it is clear that the variant regions between the motifs in the FYVE domain are permissive to insertions and that expansions and contractions within these regions appear to be frequent. In summary, the fusion of an F-box and GTPase domain to a FYVE domain in the Euglenid FRP, suggests a PI3P-dependent membrane recruitment of a GTPase signaling protein. Extensive sequence analyses indicates that FYVE domains can accommodate insertions in regions just before the ‘turret loop’ and after the cysteine at R(R/K)HHCR + 10. Further, conservation of essential residues for PI3P interaction in FRP FYVE suggests that the domain is functional.

### 3.2 TbFRP localizes to the endocytic/exocytic region of trypanosome cells

**Fig. 2:**
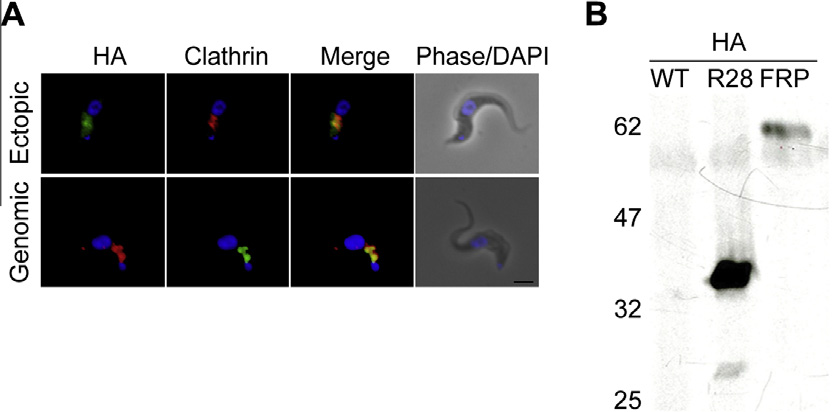
TbFRP localizes to the endosomal region of trypanosome cells. Panel A. Localization of HA-tagged full length TbFRP. Upper panels: bloodstream forms (BSF-WT) 427 transfected with pXS519-TbFRP vector were fixed with 4% paraformaldehyde and counterstained with clathrin antibodies and DAPI. HA-tagged TbFRP exhibits endosomal localization (green) and overlaps with clathrin (red) to the region between the kinetoplast and nucleus (blue). In a minority of cells overexpression of TbFRP causes an endocytic defect leading to an enlarged flagellar pocket (FP). Lower panels: BSF 427 cells genomically tagged at the C-terminus of TbFRP were fixed with 4% paraformaldehyde and counterstained with clathrin antibodies and DAPI. HA-tagged TbFRP exhibits cytoplasmic localization (green) and overlaps with clathrin (red) to the region between the kinetoplast and nucleus (blue), consistent with the overexpression data. Scale bar 2 μm. Panel B. Western blotting of trypanosome cell lines harboring various constructs and probed with anti-HA antibodies. WT represents lysate from untransfected cells, HA is HA-tagged Rab28 (Lumb et al., 2011) used as a positive control with a molecular weight of ∼26 kDa and FRP is lysate from BSF ectopically expressing C-terminal HA-tagged TbFRP. An HA-reactive antigen is detected at a migration position consistent with the full length of TbFRP at ∼62 kDa.

TbFRP was localized by ectopic expression of an HA-tagged recombinant fusion protein, as polyclonal antibodies raised against the GTPase domain failed to detect a specific signal in immunofluorescence. BSF cells were transfected with a pXS519 construct containing TbFRP with an HA-epitope tag at the C-terminus, and a cell line expressing the full length C-terminal HA-tagged TbFRP in pXS519 vector system was recovered (Fig. 2A and B). HA-tagged TbFRP migrated on SDS–PAGE with a molecular weight of ∼63 kDa (Fig. 2B) and was predominantly located between the kinetoplast and nucleus, colocalizing with clathrin in the region of the cell that contains the majority of endosomal and exocytic compartments (Brickman et al., 1995; Field et al., 1998) (Fig. 2A, upper panel), and is similar to a GFP-tagged FYVE domain (see Fig. 4C), suggesting that the FYVE-domain is important in targeting (Hall et al., 2006). However, the HA-tagged TbFRP cells developed an enlarged flagellar pocket (FP) or BigEye (data not shown), a phenotype associated with severe inhibition of endocytosis (Allen et al., 2003), and subsequently, expression of HA-tagged TbFRP became undetectable (data not shown), suggesting selection for revertants due to over-expression toxicity. This also may explain difficulties encountered in generating cell lines expressing tagged versions as multiple transfections were required to recover transformants; similar difficulties have been observed elsewhere, for example overexpression of the small GTPase TbARF1 (Price et al., 2007).

To overcome these issues associated with overexpression, an HA-epitope was added at the C-terminus of the endogenous gene (Oberhozer et al., 2006). Again, HA-tagged TbFRP clearly localized at the endocytic region (Fig. 2A, lower panel), with overlap with clathrin, suggesting an endosomal or Golgi-associated localization for TbFRP. Taken together, both overexpression and genomic tagging suggest an endomembrane localization for TbFRP and possible association with endosomal compartments, consistent with the emergence of a BigEye morphology.

### 3.3 TbFRP is not required for BSF proliferation

**Fig. 3:**
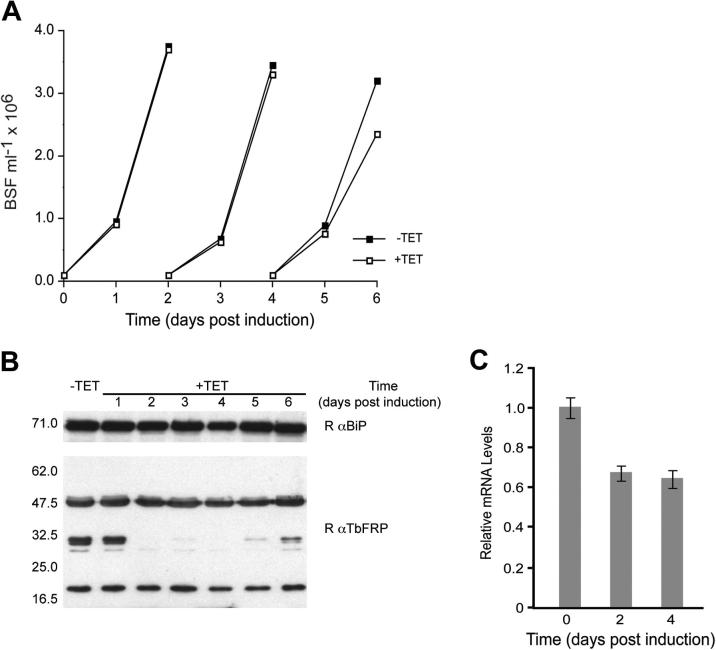
TbFRP protein is cleaved and non-essential. Panel A. Growth curve for BSF cultures transfected with p2T7-TbFRP-RNAi construct A. Cells were cultured in absence (−TET, closed squares) and presence (+TET, open squares) of 1 μg ml^−1^ tetracycline. Cells were maintained at densities below 2 × 10^6^ ml^−1^ and cell number determined every 24 h. Panel B. Loss of a triplet band is observed after two days of induction in cells cultured under same conditions as in panel A. Membranes were reprobed with BiP to confirm that equivalent amounts of total protein lysate loaded. Panel C. qRT-PCR analysis of TbFRP mRNA levels under RNAi. Data are a representative experiment of two, with error bars indicating the standard error of replicate samples. Data are normalized to β-tubulin.

To gain insight into the roles of TbFRP, we used RNA interference. Ablation of TbFRP mRNA in BSF cells did not result in a proliferative defect or disruption of the cell cycle (Fig. 3A, and data not shown), despite ∼40% depletion in mRNA levels after two days induction (Fig. 3C). This is consistent with RITseq analysis, which also suggests that TbFRP is nonessential in both culturable life stages (Alsford et al., 2011). A polyclonal antibody raised against the GTPase domain of TbFRP expressed in E. coli recognized a spectrum of bands ranging from 46.3 to 17.4 kDa, a pattern which was similar for both BSF and PCF lysates (Figs. 3B and 5). Following RNAi, three of these bands, migrating at 31.6, 31.0 and 29.4 kDa, were depleted over a period of three days, while the other bands were unaffected, suggesting cross-reactivity (Fig. 3B). The presence of a triplet of bands and their molecular weights suggests at least three closely spaced clevage sites N-terminal to the GTPase domain, and hence releasing an intact GTPase (Fig. 1A). Identical results were observed using a second RNAi construct (Fig. 1 and data not shown), suggesting that these three bands correspond to TbFRP immuno-reactivity and confirm knockdown specificity. The absence of a clear proliferative defect could be due to incomplete knockdown or a non-essential role in in vitro cultivated trypanosomes. The near complete loss of the protein triplet argues for the latter interpretation.

The detection of 31.6, 31.0 and 29.4 kDa bands likely corresponding to TbFRP suggests extensive post-translational modification. We considered the possibility that the TbFRP gene is a sequencing artifact representing two genes, one a FYVE domain protein and the other a conventional GTPase. We excluded this possibility as PCR amplification of TbFRP from genomic DNA and resequencing revealed the full length ORF at 63.8 kDa, with no internal stop codon (data not shown). This was further confirmed by ectopic expression (Fig. 2), and the identification of similar, complete ORFs, with conserved organization and synteny in additional trypanosomatids, together with the distantly related Euglenozoa, all supporting a single FRP gene. Since there was no evidence for full-length protein by Western blot using the polyclonal antibody, but that full-length protein was detected using a C-terminal tag, this suggests rapid cleavage of full length TbFRP, and release of the GTPase domain.

In conclusion, TbFRP appears to be non-essential to BSF trypanosomes in in vitro culture. Comparisons between C-terminally tagged protein and detection using polyclonal antibodies directed toward the GTPase domain suggests rapid proteolysis of TbFRP, with release of fragments corresponding to an intact GTPase.

### 3.4 The FYVE domain of TbFRP binds to phosphatidylinositol 3-phosphate (PIP)

**Fig. 4:**
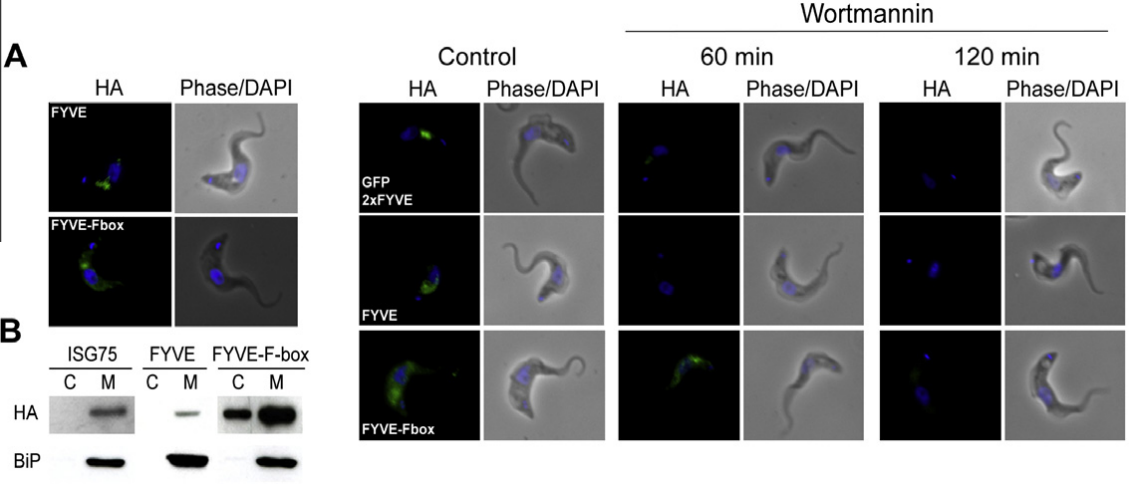
The TbFRP FYVE domain binds to membrane phospholipids. Panel A. The FYVE domain alone from TbFRP was C-terminal HA-tagged and expressed in BSF cells and localized by immunofluorescence. The protein (green) locates in the endocytic region, between the kinetoplast and the nucleus (blue), similar to the full length protein. A similar construct containing both the FYVE and F-box domains is also localized to the same region of the cell. Panel B. Cells were subjected to hypotonic lysis followed by fractionation and the cytosolic (C) and membrane (M) fractions analyzed by Western blotting. Tagged proteins are quantitatively retained in the membrane fraction except for TbFRP FYVE-F-box in which ⩾60% is membrane associated. Approximately equal levels of protein were recovered in the supernatant and pellet, confirming lysis (data not shown). Panel C. Wortmannin perturbs TbFRP FYVE domain membrane association. Trypanosomes expressing GFP2xFYVE, HA-tagged TbFRP FYVE domain or FYVE domain plus F-box domain were exposed to 3 μM wortmannin for 2 h. Aliquots were withdrawn (0, 1 and 2 h) and immediately processed for immunofluorescence microscopy. The cells were visualized under identical exposure conditions and show delocalization of GFP2xFYVE, TbFRP FYVE and TbFRP FYVE-F-box constructs.

To investigate potential roles for the FYVE domain, C-terminal HA-tagged TbFRP truncations containing the FYVE domain plus F-box and the FYVE domain alone were expressed in BSF cells using the pXS519 vector. By immunofluorescence, both constructs localized to the posterior region of the cytosol, between the kinetoplast and nucleus (Fig. 4A).

To investigate if the TbFRP FYVE and TbFRP FYVE-F-box truncation proteins bind membrane, subcellular fractionation following hypotonic lysis was performed. Membrane and cytosolic fractions were analyzed by Western blot, with an HA-tagged invariant surface glycoprotein 75 (ISG75) (Leung et al., 2011) and TbBiP (Bangs et al., 1993) as controls. ISG75 is a trans-membrane protein and BiP an ER-luminal protein; neither is expected to be released on hypotonic lysis. HA-tagged ISG75 and TbFRP FYVE were retained almost exclusively in the membrane fraction, while for TbFRP FYVE-F-box, over 60% was in the membrane fraction (Fig. 4B), an indication of substantial membrane-association. As expected, BiP was also retained in the membrane fraction in all cases. Coomassie staining following SDS–PAGE indicated an approximate 50% release of protein into the soluble pool, also consistent with efficient lysis (data not shown). Therefore, the TbFRP FYVE domain alone is capable of directing membrane targeting.

FYVE domains specifically bind to PI3P membrane phospholipids (Gaullier et al., 2000; Gillooly et al., 2000; Hayakawa et al., 2007) and previous studies have shown that wortmannin, an inhibitor of PI-3 kinase Vps34, inhibits PI3P synthesis in trypanosomes as well as higher eukaryotes (Hall et al., 2006). To determine if TbFRP FYVE binds PI3P, cells expressing HA-tagged truncations of TbFRP FYVE, TbFRP FYVE-F-box and GFP-2xFYVE (Hall et al., 2006) were treated with 3 μM wortmannin for two hours. Prior to addition of wortmannin, all three HA-tagged proteins localized to similar regions of the cell as observed for GFP2xFYVE (Fig. 4C). After one hour of wortmannin exposure, fluorescence from TbFRP FYVE and GFP2xFYVE expressing cells was diffuse; a similar effect was observed after two hours for the TbFRP FYVE-F-box expressing cells (Fig. 4C). The data suggests that reduction of PI3P in membranes due to wortmannin inhibition of TbVps34 resulted in a failure to recruit GFP2xFYVE and TbFRP FYVE to endosomal membranes. In addition, the cells developed an enlarged flagellar pocket (FP), a phenotype also obtained by silencing of TbVps34 (Hall et al., 2006), confirming inhibition of TbVps34 function. In conclusion, these data suggest that the FYVE domain of TbFRP is a functional PI3P-targeted membrane-binding domain, while in these assays the F-box appears to contribute minimally to targeting.

### 3.5 Developmental and density-dependent expression of TbFRP protein

**Fig. 5:**
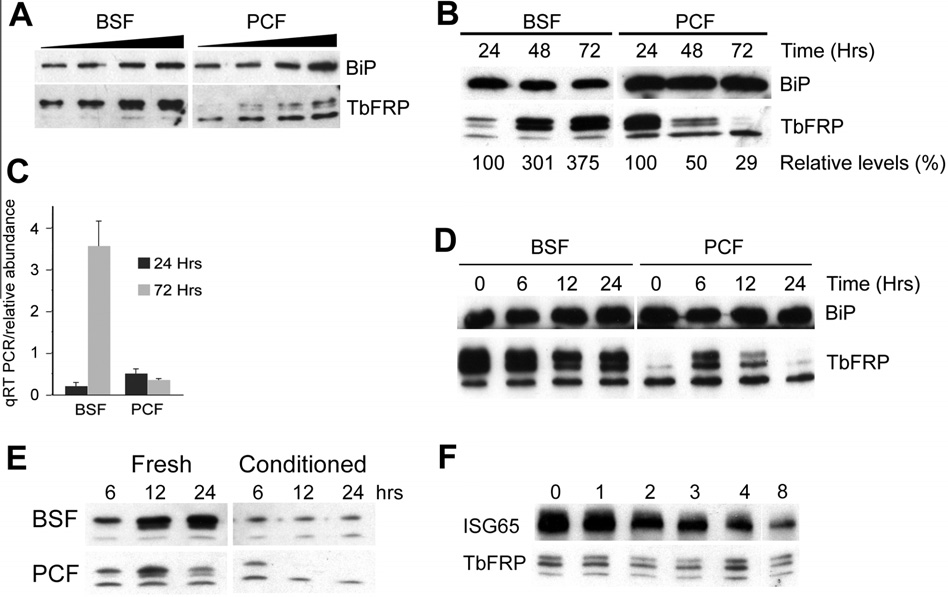
TbFRP expression is cell density dependent. Panel A. Equivalent but increasing amounts of BSF and PCF protein lysates (shown as black wedge) probed for TbFRP by Western blotting. BiP was used as a loading control and the same blot probed after stripping. Panel B. BSF and PCF cells were cultured at a starting density of 1 × 10^5^ and 1 × 10^6^ ml^−1^, respectively, and maintained continuously for 72 h. Aliquots of both cultures were withdrawn after every 24 h and probed for TbFRP by Western blotting and the relative levels of the TbFRP triplet determined (numbers below). Panel C. A bar graph of the TbFRP mRNA levels in BSF and PCF at 24 and 72 h cultured as in panel B. The experiment has been repeated three times with essentially identical results. Panel D. Western blot of cells taken at 72 h from cultures in B, washed and cultured in fresh media for 24 h. Aliquots were analyzed by TbFRP Western blotting after 6, 12 and 24 h. The experiment has been performed three times with essentially identical results. Panel E: Effect of conditioned media on TbFRP level. Trypanosomes cultured for 72 h without passaging are washed and subsequently cultured for 24 h in fresh and conditioned media. Aliquots were analyzed for TbFRP levels after 6, 12 and 24 h. The experiment has been performed three times with essentially identical results. Panel F. Turnover of TbFRP in BSF cells. Cells were treated with cyclohexamide and at the times indicated (hours) aliquots withdrawn and lysates prepared. Following SDS–PAGE and Western blotting the levels of ISG65 and TbFRP were determined. TbFRP is more stable than ISG65.

The loss of the protein triplet observed on TbFRP silencing (Fig. 3) indicates that these polypeptides are encoded by the TbFRP gene. Potentially developmental expression at the protein level may correlate with slight upregulation observed by qRT-PCR (about 1.5 fold, data not shown). By semi-quantitative Western blotting, using TbBiP as a loading control, we observed that overall reactivity towards the TbFRP triplet was significantly higher in BSF compared to PCF cells, and that the relative intensities of the three bands is distinct, with a clear prominence of the lower 29.6 kDa band in the PCF lysates (Fig. 5A). Further, we also noticed that the overall expression level and relative levels of the three bands appeared to vary depending on cell density within cultures.

To investigate this directly, cultures of BSF and PCF cells, at starting densities of 1 × 10^5^ ml^−1^ and 1 × 10^6^ ml^−1^, respectively, were cultured in fresh media for three days without sub-culturing and aliquots removed every 24 h for analysis of protein level by Western blotting and mRNA by qRT-PCR. In BSF cultures, the relative levels of the 31.6 and 31.0 kDa bands increased while the 29.4 kDa band remained unaltered during proliferation and with increased cell density (Fig. 5B); overall reactivity of the triplet increased threefold. In PCF cultures, the intensities of the 31.6 and 31.0 kDa bands decreased significantly, with the 29.4 kDa band remaining at unaltered levels. Levels of TbFRP mRNA transcripts were also quantified by qRT-PCR in unrefreshed cultures. In BSF cultures TbFRP mRNA levels increased by over fivefold after 72 h, while in PCF cultures there was no significant change (Fig. 5C).

To determine if TbFRP expression is influenced simply by cell density or a result of trypanosome conditioning of the media, cells cultured for 72 h as above (BSF and PCF cell densities of 3.6 × 10^6^ and 2.7 × 10^7^ ml^−1^, respectively) were harvested, washed in fresh medium and then returned to fresh medium for 24 h. Aliquots were removed after six, 12 and 24 h and TbFRP protein levels determined. In BSF cells the protein levels exhibited a slight decrease (Fig. 5D). In PCF cells an increase by six hours in fresh medium, followed by a rapid decrease was observed, likely due once more to an increase in cell density in the cultures. This may suggest that a factor released by the cells affects expression of TbFRP; at six hours levels are low and the increase in TbFRP is consistent with the data in Fig. 5E, but following a significant increase in cell density at 24 h, TbFRP levels decrease.

To examine the effects of media, cells cultured for 24 h were harvested, washed appropriately and cultured either in fresh media or 72 h conditioned media. The level of TbFRP in BSF cells cultured in fresh media increased, consistent with Fig. 5B (Fig. 5E). However, in conditioned media, TbFRP levels remained unchanged, though an increase was expected if factors secreted into the media were involved in TbFRP copy number regulation in BSF. For PCF cells in fresh medium, TbFRP levels slightly increased (12 h), before decreasing possibly due to increased cell density. In conditioned medium TbFRP levels significantly decreased as expected. Further, in fresh media we found that TbFRP was a comparatively stable protein, as cycloheximide-treated cultures turned the protein over less rapidly than ISG65, which has a half life of ∼five hours (Fig. 5F) (Chung et al., 2004). This suggests that the rapid loss of TbFRP in cells transitioned to conditioned media is due to accelerated degradation, and not decreased rates of synthesis.

These data demonstrate several aspects of TbFRP expression. First, that the relative intensities of the silencing-sensitive triplet of bands are distinct between BSF and PCF, suggesting developmental variation in TbFRP processing. Second, the level of expression is sensitive to the density of the culture, and that this appears to be, at least in part, mediated by a soluble factor released into the media by the cells. Finally, in BSF cells levels of TbFRP mRNA and protein both appear to be density dependent, suggesting that in this life stage at least, that control of TbFRP is likely mediated via alterations in mRNA turnover as well as protein level control.

## 4 Discussion

The mechanisms co-ordinating environmental sensing, life cycle progression, developmental regulation and modulation of gene expression remain poorly understood for pathogenic trypanosomes, and even more so for Euglenid protozoa. These organisms all possess substantial repertoires of kinases, GTPases and phosphoinositide kinases (Field 2005; Hall et al., 2006; Parsons et al., 2005; Koumandou et al., 2008), three of the major signal transduction protein families known from higher eukaryotes. However, the absence of promoter-driven gene expression has resulted in a disconnection between potential stimuli and the molecules that populate such pathways. A comparatively well characterized pathway is known to mediate differentiation of BSF trypanosomes, and which clearly involves reversible protein phosphorylation (Szöor et al., 2010). Here we have examined in T. brucei a highly unusual small GTPase, TbFRP, which also bears a FYVE domain and an F-box, and which appears to be restricted to the Euglenozoa. Our data suggest that fusion of a possible Golgi-associated Rab-like GTPase domain, Rab18, occurred at the root of the Euglenid clade, to create FRP. Hence, from this deep taxonomic representation it is likely that TbFRP and its orthologs have a fundamental function, rather than one specific to parasitic species.

TbFRP is a 63.8 kDa protein that is targeted to the endomembrane-rich region of the cell. The protein appears to be rapidly cleaved, with release of the C-terminal GTPase domain as a complex triplet of bands migrating between 31.6 and 29.4 kDa. We presume that this is an extremely rapid process as it was very difficult to detect full-length protein using antibody against the GTPase domain in whole cell lysates or by metabolic labeling and immunoprecipitation (data not shown). Evidence that the triplet is indeed derived from TbFRP comes via selective depletion following RNAi. We also show that the FYVE domain is functional, and can bind PI3P, with a localization that is highly similar to that obtained previously for a FYVE construct, suggesting endosomal targeting (Hall et al., 2006). The role of the F-box is unclear, but we note that this domain is frequently involved in rapid protein turnover, for example in mitosis via interaction with the SCF ubiquitin ligase, and that F-box proteins have also been found to be involved in dynamic signal transduction pathways in a wide range of organisms (Chico et al., 2008; Jonkers and Rep, 2009; Shishova and Lindberg, 2010; Cheng and Li, 2012). The F-box therefore may be coupled to the rapid proteolysis of TbFRP.

Silencing of TbFRP had no significant effects on proliferation, although we found great difficulty in our attempts to overexpress the full length protein, with an extremely low frequency of transformants (data not shown). Even in those cases where transformants were obtained, cells lost TbFRP expression over a period of <50 generations, with suggestions of defects to endocytosis manifest as an enlarged flagellar pocket. Therefore it appears that regulation of the level of TbFRP protein is important to trypanosomes with overexpression being toxic, and potentially having a negative impact on endocytosis, while TbFRP may not contribute substantially to cell physiology in in vitro culture; overexpression toxicity is likely due to excessive sequestration of PI3P by the FYVE domain, as observed previously (Hall et al., 2006), and potentially explains the difficulty in obtaining transformants. Further, we observed a density-dependent production of FRP GTPase-containing proteolytic fragments, and that the increased copy number of these cleaved products was accompanied by a five-fold increase to mRNA transcript levels in BSF trypanosomes. TbFRP RNA has a comparatively short half life at under ten minutes and is present at rather low copy number (approximately one mRNA per cell), and which may contribute to the dynamic behavior of TbFRP, and also explain why silencing had little impact (Manful et al., 2011). It is unclear how this process relates to life cycle progression, although we can speculate that perhaps the growth arrest we observe on overexpression is coupled to a density-dependent role via increased TbFRP levels, where potentially increased TbFRP FYVE domain would sequester PI3P, and potentially could contribute to transient remodeling of trafficking and other pathways (Natesan et al., 2007). Here, high levels of TbFRP could be associated with stumpy forms and procyclics, for example. Further, the presence of the F-box implies a connection to the ubiquitylation system and rapid turnover, and hence a dynamic TbFRP population.

## 5 Conclusion

TbFRP is a multi-domain GTPase present throughout the Euglenozoa. The presence of a FYVE domain, an F-box and a GTPase is, as far as we are aware, a unique combination, and together these domains suggest endosomal targeting, together with rapid turnover. TbFRP may act to integrate endosomal function and phosphoinositide signaling in a highly dynamic manner.

## 6 Authors’ contributions

MCF and VOA conceived the experiments. VOA carried out the experimental. MCF and VOA performed the informatics. MCF and VOA analyzed the data and wrote the manuscript.
