# Supplementary material for: TbFRP, a novel FYVE-domain containing phosphoinositide-binding Ras-like GTPase from trypanosomes
Source: Exp Parasitol. 2013 Mar;133(3):255–64. doi: 10.1016/j.exppara.2012.11.007 (PMC3593210; doi:10.1016/j.exppara.2012.11.007)
Supplement: Supplementary data 1 — Supplementary data. [file mmc1.pdf]

```
MUSCLE (3.8) multiple sequence alignment


EgracilisZ1        ------------------------------------------------------------
Tborrelli          -------------------------------------------------------MDHVD
Pserpens           ---------------------------------------------------MSTDRQDVS
Lbrasiliensis      --------------------------------------------------MEKDMLSLYP
Ltarantolae        ---------------------------------------MEGPRTTHGDTSEKEMLSPCS
Lmexicana          ------------------------------------------------------------
Lmajor             ------------------------------------------------------------
Linfantum          ------------------------------------------------------------
Bsaltans           -------------------------------------------------------MDHAD
Tvivax             -------------------------------------------------------MDYMD
Tcongolense        MVKGASSIIFFFRSPLLKVFLGSTAGRKKRNRHLSNTGRIAAACALLAGYARQDLMDHID
Tbrucei            -------------------------------------------------------MDHID
Tbgambiense        -------------------------------------------------------MDHID
Tcarassii          -------------------------------------------------------MDHVD
Ttheileri          -------------------------------------------------------MDHVD
Tgrayi             -------------------------------------------------------MDHVD
Tcruzi             -------------------------------------------------------MDHVD


EgracilisZ1        ------------------------------------------------------------
Tborrelli          LTVGSD---SEFLTAISLRMP---------------------------------------
Pserpens           LVSSVNNGEDEPSMTVSTSGPSGRMPRVISAVSSVSKEFLPGDIVSGGVGLKNLTTSSYI
Lbrasiliensis      SHLQERRASGPPVLHVNVLCG---------------------------------------
Ltarantolae        NHIHERRSCGPPVLRVHVLRG---------------------------------------
Lmexicana          ------------------------------------------------------------
Lmajor             ------------------------------------------------------------
Linfantum          ------------------------------------------------------------
Bsaltans           IAASPELTGG-PLTQLHVRIP---------------------------------------
Tvivax             FASARETGPLLSPATISVSIP---------------------------------------
Tcongolense        ITEVRELGGEVSLKTLNISIP---------------------------------------
Tbrucei            LAEEREMGNGDPIMVLNVSVP---------------------------------------
Tbgambiense        LAEEREMGNGDPIMVLNVSVP---------------------------------------
Tcarassii          VVGTREMDSASVQRTLHVTIP---------------------------------------
Ttheileri          LASAREMGSSPAFTTLHVSVP---------------------------------------
Tgrayi             VAGTREMGAAASLTTLHVFIP---------------------------------------
Tcruzi             VAGTRDMGSTPSLTALQVIIP---------------------------------------


EgracilisZ1        ------------------------------------------------------------
Tborrelli          --------AFANSKG---------------------------------------------
Pserpens           VSAGDTQALGDSLHPSTSAIIPPSSSQRRSLDTNITERYYPFNATRHPIRTLSTSVSLNA
Lbrasiliensis      --------LSNEDITSSSFVA----TIGR-SSTGAGTLNRVNPTSSIPASATAFASRRAR
Ltarantolae        --------MPKGDTTSDSFSS----TFDRTTSTGTGSPNRAGPLSSLPAS----ASRRTS
Lmexicana          ------------------------------------------------------------
Lmajor             ------------------------------------------------------------
Linfantum          ------------------------------------------------------------
Bsaltans           --------FNAAGDGGGSSAG---------------------------------------
Tvivax             --------YTTAAVSSSSSNT--------------------------------CNSSVSG
Tcongolense        --------FTSSSSPRNDSDG---------------------------------------
Tbrucei            --------YSANSSDRKSNVS---------------------------------------
Tbgambiense        --------YSANSSDRKSNVS---------------------------------------
Tcarassii          --------SSKNCSGHFPSVT---------------------------------------
Ttheileri          --------YSSNNSTYNSNSN---IYNNNNNNSSHINNNSNASGSTSGVAAYGGSTSPRT
Tgrayi             --------YSNSATNKTSNSG-----------------------AAGVPAVSSSFTPRTA
Tcruzi             --------YGSNSNNNNNNNN----------------------NNNNNFYSGGVCFTRCP


EgracilisZ1        --------------------------------------LKPVSEWVQDDVVTHCVS--CH
Tborrelli          -------------------LGGGFTALRPPRTN--DVYCMPREKWIPDARVVECMASECR
Pserpens           MNPPQLLGLGAIFSSSVHPQNGDARSFAKPTLDPASVRWCSPDHWVPDAQVVNCMAPDCK
Lbrasiliensis      NGSLSPLRSFPSSSVANGTSPVQSSTLAKPVLDLSQVRCIPIDQWIPDAHVVNCMAPDCG
Ltarantolae        DGVQSTMGPAVNACSTGAAGIVQSSTLAKPVLDLSQVRCIPLDQWVPDANVVNCMAPDCS
Lmexicana          ---------------MSGTGVVQSSTLAKPVLELSQVRCIPLDQWVPDAHVVNCMAPDCS
Lmajor             ---------------MSGNGAVQSPALAKPVLDLSQVRCIPFDQWVPDAQVVNCMAPDCS
Linfantum          ------------------------------------------------------------
```

```
Bsaltans           --------RYHQRTNSTNPALYSRQTIAKPTLNRTVV-LMPSSRWIADNQVACCTAPECQ
Tvivax             NGNDNGVTSSTCLSSSGTIASHKFTRLTKPTLDPSHIRFCPPSQWVPDAQIMTCMTHECN
Tcongolense        ------GTNGDSKTIGLSTSATTW--LTKPVLDPWRICLCPIKRWVPDMQVTNCMAHECN
Tbrucei            -------SPLLSKCASKQTSGVMW--LTKPTLDPSCIRLCPLRRWVPDTQVSTCMAHGCN
Tbgambiense        -------SPLLSKCASKQTSGVMW--LTKPTLDPSCIRLCPLRRWVPDTQVSTCMAHGCN
Tcarassii          -------------NLSPPQSSSLQRTLAKPTLDPSCIRFCPLDRWVPDAQVIACMAPGCN
Ttheileri          PHVHSLAGSMAASSVMNNPTSNTSRSLAKPTLDTSHIRLCSEECWMPDAQVMTCMAHECN
Tgrayi             TSAGTAVASSAALNGGGTTASSRWM-LAKPTLDPSCIRFCPETCWVPDAQVMTCMAHDCN
Tcruzi             SLTANVTRSLSVSCGGDGSSFSNVWSLAKPTLDPSHIRFCPEGCWVPDAQVMTCMAHECN


EgracilisZ1        AAFSIFNRKHHCRVCGHIFCHFCSAKTLPM------------------------------
Tborrelli          QQFTFFTRRHHCRVCGRIYCAKCSSESIHR-----------YVDTNFTQRSDVNYVQLSA
Pserpens           MPFSIFTRKEHCKLCGRVFCYSCCSNEVEILTDGSPCSSHSRNASGNIKGSEMLSSNGFP
Lbrasiliensis      NSFSLFNRKHHCRMCGRVFCSSCCNNLVYIPAAVAQNAASSSTDGMAMGSCTATSYTPVS
Ltarantolae        NSFSLFNRKHHCRMCGRVFCSSCCNHLVSIPAVVVSRANGSPEG-MAVGGYVATSNASII
Lmexicana          NSFSLFNRKHHCRMCGRVFCSSCCNNLVYIPAAVVNRANSSTEGPAAV-SYVATANTSMS
Lmajor             NSFSLFNRKHHCRMCGHVFCSSCCNNLVYIPAAVVNKANSSTEGAAAAGSYSAMTNTSLS
Linfantum          ------------------------------------------------------------
Bsaltans           LPFTIFNRKHHCRVCGNIFCANCSSHTLPA------------------------------
Tvivax             VFFSFFNRRHHCHICGRVFCSACCSRMVNL-------------------GDYPRMSSPNS
Tcongolense        VAFTMFNRRHHCRLCGRIFCFACCSNTVRV---------------PSGPHLAASHYAGAD
Tbrucei            VAFSMFNRRHHCRVCGRVFCSACCSETVNA----------------LVQSALEVQSNPIE
Tbgambiense        VAFSMFNRRHHCRVCGRVFCSACCSETVNA----------------LVQSALEVQSNPIE
Tcarassii          VSFSLFNRRHHCRICGHLFCAACCSSHTVTV-----------------PSTASVAMSGRSS
Ttheileri          VSFSLFNRRHHCRVCGRIFCAACCSHFITL-----RGDNHTGTTGSLVTSFLDGVESPTA
Tgrayi             VSFSLFNRRHHCRVCGRIFCAACCSHIITA--LNYHNNNDSYYSQNQRQLQNSSYTNSMA
Tcruzi             VSFSLFNRKHHCRLCGRLFCAACCSHVITI---------------RSTRGGLSASCALSG


EgracilisZ1        ------------------------------------------------------------
Tborrelli          EDDHHQHLAADT-----ETAPNGGPLVSSSSTTVRSIADNNINSNNI-----NSNNRKTS
Pserpens           HPRSHSQIANET-----GSNPVTGSSSYPKRSESTGFSNVTTVHTAMGNAVAISTSATSN
Lbrasiliensis      SAITSELQQFHRACARDGSAPSNGMRRGSQSPITSAPDLFPATSMTLHHGSLVVCGEGNG
Ltarantolae        PAATSEPQSLNRASAGEGSASSTGMRRGPQAQVAQGSESFFASSATLLSGTLPVSSDGNG
Lmexicana          SAATPEPQQFNRACASDGSASSNGMRRGSQAHAAQGADVSSASSTTLFPGPLHVCNDGNG
Lmajor             SVVTPEPQQFNRACASDGSALPNGMRRGPQAPAAQGLDVSSASSSALLPGPLPVCSDGNG
Linfantum          ------------------------------------------------------------
Bsaltans           -VGGAEDAAVNK------------------------------------------SIAAAQ
Tvivax             RSDGMNGVHLNA------VAPQSSVRTSVASSIFSPR---------------YAPQREFQ
Tcongolense        NARGVDRMPLPN-----GEAVAREAGGRDDS----------------------LDQNKQN
Tbrucei            ACGGVDKTSLPS-----GRVSGTDIANGEDN---------------------ISQQQQQG
Tbgambiense        ACGGVDKTSLPS-----GRVSGTDIANGEDN---------------------ISQQQQQG
Tcarassii          PCHGPPKNLSRR-----NTAPGTLPAQPTASPVQTSTASS------------MSCISAAT
Ttheileri          SSTTPHRIGDKVHGQRNSILSSTRPLQSSPSQSLQQIGSS------------YHQRQPSQ
Tgrayi             QCTVSCGDALDSF----GSPSTVQSHRGDGSPLGACST--------------PSSARLHQ
Tcruzi             AEGQETATSLPA-----ILGATDDNSAGGASSNPNPSSFYPSQA--------QLVQQQQH


EgracilisZ1        ---------PAPSY----------------ESVRVCDNCHADHQLAIVKNDFERKVENAR
Tborrelli          PPS---MASPLSPV---------------TNEMRVCKECYYSYQLAICRRDVATGLPRRR
Pserpens           PSC---AASVDNVL----SMGTPLPQTSKGTLYRVCNECFYETQLVVSVRG-EDGSPRRK
Lbrasiliensis      NLP---SSALNSVVAMTTAPSPSSPSTQTSVPCRVCASCSYEVQLVVSTRQ-ENGEPRRR
Ltarantolae        HLS---FNSLTAPS---------------SVPCRVCSGCSYEIQLVVSARQ-ENGEPRRR
Lmexicana          HLTSNALTSPSTVTAVTTLPSSLSPSTQAAVPCRVCASCSYEVQLVVSTRQ-ENGEPRRR
Lmajor             HPPSNALTAPSAVT---ALPSSMSPSTQAAVPCRVCASCSYEVQLVVSTRQ-ENGEPRRR
Linfantum          ------------------------------------------------------------
Bsaltans           QVA---ANNNSNAS----------------TTVRVCAECFYEHQLVVVRRV-NEGVLRRR
Tvivax             SQQ---LACSTSVP----------------GSCRICTSCHYETQLVVSRRG-PNGGLRRK
Tcongolense        QPQ---QVDYTTMT-----------------AYRICTSCHYEIQLVIPRRD-CNGEVRRK
Tbrucei            QVQ---FMNPTTVA-----------------AYRVCFACHYEVQLVVSRRD-RNGEVRRK
Tbgambiense        QVQ---FMNPTTVA-----------------AYRVCFACHYEVQLVVSRRD-RNGEVRRK
Tcarassii          TTT---TTATAPTP----------------STYRICSSCNYEIQLVVARRD-ENGEVRRK
Ttheileri          QQQ---QLQPSGSS-------------SFSTTHRICSACYYELQLVVSRRD-HTGEARRK
Tgrayi             QQQ---FVTSSPTT-----------------TCRVCSACHYELQLVVPRRD-HNGEARRK
Tcruzi             STS---HLEPTATT----------------TTHRICSACYYEIQLVVPRRD-QNGEVRRR
```

```
EgracilisZ1      DRGALACLSREVMERVLAFLPAPWLVHVAPLVSRDFYFLARSNALWKPLYEARWRGLPV-
Tborrelli        CRGELKMIQRARIVDICSFLSLKDLFNVS-LVSSDFYFITRDNNLWVYFNAMRWEGYNNN
Pserpens         CRGQLKMFQRALLVNVLSFLSLRDLLETA-LVSSDFYFMSRDNVIWYQYNTARWLKETEN
Lbrasiliensis    SRGELKMIQRVLLVNIMTFLTLRDLANVS-LVSADFYFMSRDNIIWYQYNMTRWAQESE-
Ltarantolae      SRGELKMIQRVLLINVMSFLTLRDLANVS-LVSADFYFMSRDNIIWYQYNMTRWAQEAE-
Lmexicana        SRGELKMIQRVLLVNVMSFLTLRDLVNVS-LVSADFYFMSRDNIIWYQYNMTRWVQEAE-
Lmajor           SRGELKMIQRVLLVNVMSFLTLRDLANVS-LVSADFYFMSRDNIIWYQYNMTRWVQEAK-
Linfantum        ------------------------------------------------------------
Bsaltans         ARGHLKMFQWPLIVNILSYLSLSELLCVS-AVSSDFYFMSRDNQLWFTHNMQQWGASMA-
Tvivax           CRGELKMIQWSLLVRILSFLPMEDLLEVS-LVSSDFYFMSRDNVIWLRHNMSRFAMEKE-
Tcongolense      CRGELKMLQRLLLVRIVSYLSMEDLLKVS-LVSSDFYFVSRDNVIWYRYNMTRCVREEE-
Tbrucei          CRGELKMLQWSLLVRVLSYLTMEELLGVS-LVSSDFYFMSRDNVIWYRYNMTRCLREEE-
Tbgambiense      CRGELKMLQWSLLVRVLSYLTMEELLGVS-LVSSDFYFMSRDNVIWYRYNMTRCLREEE-
Tcarassii        CRGELKMLQWPLLVRMLTYLSRPDILQVS-LVSSDFYFMSRDNLIWYHYNMARCKEEEL-
Ttheileri        CRGELKMLQWSLLVNMLTYLSMHDLLEVS-LVSSDFYFMSRDNVIWYQYNLAQYQQKEK-
Tgrayi           CRGELKMMQWPLLVRMLSYLTMHDLLEVS-LVSSDFYFMSRDNLIWYQYNMARCWQEVE-
Tcruzi           CRGELKMFQWSLLVKVLSYLSMHDLLEAS-LVSSDLYFISRDNVIWYQYNMAQRRQKEV-


EgracilisZ1      ------------------------------------------------------------
Tborrelli        NGERTSKERTEEDTRSSTPTSIGSYG-----------------YRP--------------
Pserpens         PYFSFTGDTNHMLTVAAANSASASGGIRYWFSAHDNYYKSSSPSASSALTIALDGSSASV
Lbrasiliensis    ------LPRLSSLKSHAEASRTQQQKGLSWYASSSGSAARSSLAED--------------
Ltarantolae      ------LPRLSTLNSRAAASQMQQQRAPSWYAYSSVSGVLNSFADD--------------
Lmexicana        ------LPRLSSLNSRAAASRMQQQRGPSWYTYSSGNVARSSLIDD--------------
Lmajor           ------LPRLSSHNSRAAASRMQQQRGPSWCTYSSGSGPRSSLVDD--------------
Linfantum        ------------------------------------------------------------
Bsaltans         ------QEKTTTTRKGSNATAAST-------------------AAQ--------------
Tvivax           ------IQRTVMKTVEGTSALLKQNS-----------------HGR--------------
Tcongolense      ------VQRMLTMTVESPNSTRKWRS-----------------TGK--------------
Tbrucei          ------LQRMLSTTLGSRNSTRRQRL-----------------PQK--------------
Tbgambiense      ------LQRMLSTTLGSRNSTRRQRL-----------------PQK--------------
Tcarassii        -----------QLKLPLALPSLRRLR-----------------SLT--------------
Ttheileri        ------ELKIPTASNYFNPSLRHRLLN---------------------------------
Tgrayi           -------LKIPTTRSSSSSFSLRRRL------------------LN--------------
Tcruzi           ------QLRIPPRRQNSSLSLRRRFL-----------------QRQ--------------


EgracilisZ1      -------------------------NPQQTNSRETNWVFYQTYQRQHLSDAVMSNSRLRM
Tborrelli        -----ISRDTSATGFLKGVTPSDGGE--ISNHCRYNYTQFLQYCTLLENARCGGLANFAT
Pserpens         VEGSIKFPRAPVLEDATVLTQGEAFKRVISHHARYNYTQFLDFARRQEMARCEGLAWFSV
Lbrasiliensis    -----MFNSAPVIQDAAALSESEAAKRVISLHARYSYTQFLDFARRQEMARCEGLSSFSL
Ltarantolae      -----MFSSAPVIQDATALSESEAAKRVISLHARYNYTQFLDFARRKEMARCEGLSSFSL
Lmexicana        -----MFPSAPVIHDATALSESEAAKRVISLHARYNYTQFLDFARRQEMARCEGLSSFSL
Lmajor           -----MFSSAPVIQDATALSESEAAKRVISLHARYNYTQFLDFARRQEMAWCEGLSSFSL
Linfantum        -------------------------------------------------MARCEGLSSFSL
Bsaltans         -----KGGKQALVVS----VAAPLSKVVISHHARYNYTQFLDFARRLEGARCQGLSSFSA
Tvivax           -----QRREIPFSTDIDDIVSADALKPSISLNARYNFTQFLDFTRRQETTRCKGLSCFAV
Tcongolense      -----ITRELSFNTDFDSITSADAAKPAISLHARYNFTQFLDFTRRREATRCKGLSCFSV
Tbrucei          -----QLGELTFGTDFDSITSAGAAKPTISLNARYNFTQFLDFTRRREATRCKGLSCFSV
Tbgambiense      -----QLGELTFGTDFDSITSAGAAKPTISLNARYNFTQFLDFTRRREATRCKGLSCFSV
Tcarassii        -----RQNQKIVNTDLDGVAADDAAKRVISLHSRYNFTHFLDFARRHEMSRYKGLSSFAV
Ttheileri        -----RHKVRAFRTDFEDITPTDASKRVISLHARYNFTQFLDFARQQEMARCKGFLSFSV
Tgrayi           -----RAKPQTFLTEFGDIIPTDASKRVISLHARYNFTQFLDFARRRELARCKGVSSFSE
Tcruzi           -----KEPLRAFGIEFGDVSSSDASKRVISLHARYNFTQFLDFARRQEMARCRGLSSFSV
                                                                     .   :

EgracilisZ1      RIQTLLTGSLKFFLIGASGVGKTCLLHRFL-TGEYRRQPSGTVGVAV-------------
Tborrelli        NAKLLLMKSVKVAVIGHSRVGKSVMVRHFMDELYGSIRGNTRIFSPTHASHHRRTTSHTT
Pserpens         AARVLFSSTIRVALIGPPGVGKTTSLLTFL--------KKNKRGSTV-------------
Lbrasiliensis    GARILLSSPIRVALVGPCGIGKTASVRAFL--------GEKLSQMVV-------------
Ltarantolae      GARILLSSPIRVALVGPCGIGKTASAHTFL--------GEKPSQMVV-------------
Lmexicana        AARILLSSPIRVALVGPCGIGKSACAHAFL--------GEKPSQMVV-------------
Lmajor           GARLLLSSPIRVALVGPCGIGKTASAHAFL--------GEKPSQMVV-------------
Linfantum        GARLLLSSPIRVALVGPCGIGKTASAHAFL--------GEKPSQMVV-------------
Bsaltans         GAKQLLSSPIKICVVGPPGIGKTFMMKRFTGEISGDPTTIASHALSI-------------
Tvivax           GAHTLLSSPLKVALIGPCGARKTLMMQHLVPFNQHNGRNAGSEHSET-------------
Tcongolense      STRTLLSSPIKIALIGPSGVGKTMLMREWV--QGGRPDTDGTSYEAS-------------
```

```
Tbrucei            GMRDLLSSPIKIALIGPCGVGKTSLMREWG--RQRRPGDNSARFAVT-------------
Tbgambiense        GMRDLLSSPIKIALIGPCGVGKTSLMREWG--RQRRPGDNSARFAAT-------------
Tcarassii          CARMLLSSPVKIAIIGPSGIGKTPMLQHFI---------GTIPLDGP-------------
Ttheileri          GARMLLSSPLKIAIIGPTGIGKSSMVRCFA-----GPHAAHTSWREP-------------
Tgrayi             GARMLLSSPLKIAIIGPAGVGKTALVQQFV--DQHE--NRRLAGRPL-------------
Tcruzi             AARTLFSSPLKIAIIGSMGVGKTLLVRHFV--VSHE--NGGSSGGRI-------------
                     . *:  .:.. ::*     *:

EgracilisZ1        --------RTRPVQVTVGDPALSSPRCPPVSLTLAGATP-AGPVTNLVFYDASGDPRYEA
Tborrelli          AQLDKDGYR----------PTCGLVVS-HKRISITGEITLPQSGVII--YDVCGDSRYEK
Pserpens           --------H----------STMGFTRH-KVTLWTGGDSK-VGVNLEI--YDVSGDERYES
Lbrasiliensis      --------R----------PTIGFERR-AVTVRLARGLS-TEAVLHI--YDLSGADRYRE
Ltarantolae        --------R----------PTIGFERR-VVTVRLARDLS-TEAVLHI--YDLSGADRYGE
Lmexicana          --------R----------PTIGFERR-AVTVRLARGLS-AEAVLHI--YDLSGADRYGE
Lmajor             --------R----------PTIGFERR-AVRVRLARGLS-AEAVLHI--YDLSGADRYGE
Linfantum          --------R----------PTIGFERR-AVTVRLARGLF-AEAVLHI--YDLSGADRYGE
Bsaltans           --------H----------PTCGFTTY-TKRASIVGGLT-TTATFTI--MDVAGDRRYEQ
Tvivax             --------Q----------PTASFQTY-EKAIHVTGGLT-ADARLQV--FDISGEKRFRE
Tcongolense        --------V----------PAVPFNRF-EEVVHLSGGLT-ADARLQV--FDISGHPCFEE
Tbrucei            --------P----------PTVTFNQF-DKTVFLTGGLT-ARAQLRV--FDISGHPRFRE
Tbgambiense        --------P----------PTVTFNQF-DKTVFLTGGLT-ARAQLRV--FDISGHPRFRE
Tcarassii          --------R----------ATMGFTRY-CKRVHLTGGLV-SDVMLHI--FDISGEPRYEE
Ttheileri          --------L----------PTMGFTRY-EKTVHLTGSLM-ADVMLHV--FDISGEPRFEE
Tgrayi             --------L----------PTMGFTLY-EKTVHIVGSLT-TDVRLQI--FDISGEPRFEE
Tcruzi             --------S----------PKLGFVRY-AKMVHLTGSLA-ADVMLHI--YDISGELRFEE
                                      .                          :   * .*   :

EgracilisZ1        LLPLYYVGVQVACVFFDLTAPETLEVCAHWCRALQATLPPDVVIAICGLKGD--------
Tborrelli          LRALVCQSVHVIILCYNASLKKSFIEAAVQMTSIESMLGPQPVV-MCGIIDQ--------
Pserpens           LRRFVCSHCHAIGLCYNPCRKVTLVQAADVMMGVEPALGPQPVV-VCGLIPSRENGATDA
Lbrasiliensis      LRRFVCRHCHAIGLCYDPSRKMTLVQAADIMMELEGALGPQSVV-VCGLVRQPPRASSSG
Ltarantolae        LRRFICRHCHAIGLCYDPSRKVTLVQAADIMMGLESTLGPQPVV-VCGLVRPPHPSSSSG
Lmexicana          LRRFVCRHCHAIGLCYDPSRKVTLVQAADIMMGLESALGPQSVV-VCGLMRQPHHTSSSG
Lmajor             LRRFVCRHCHAIGLCYDPSRKVTLVQAADIMMGLESALGPQPVV-VCGLMRQPHHTSSSG
Linfantum          LRRFVCRHCHAIGLCYDPSRKVTLVQAADIMMGLESALGPQPVV-ICGLMRQPHYTSSSS
Bsaltans           LRGLCAQSAHAVVVCYDTHSKMSLVHAANMMTSLEPFLGPQPAV-VCGFVTD--------
Tvivax             LRRLICLHCHAIGLCYDPQSRTSFMEAVDMIQDLVSALGPQPVV-LCGVLPQAST-----
Tcongolense        LCRFICARCHIVVLCYDPRRRSSFVEAQEMMAKVDSALGAQPVV-VCGISPP--------
Tbrucei            LTRFVCASCHAVVICYDPQRKSSLMEAKAIIADVETKLGVQPVI-VCGLIPP--------
Tbgambiense        LTRFVCASCHAVVICYDPQRKSSLMEAKAIIADVETKLGVQPVI-VCGLIPP--------
Tcarassii          LRRFICSSCHAIGLCYDAQRKVTLAEAADVMNGVEPVLSAQPVV-VCGIVPPRREHSPPA
Ttheileri          LRRFICSNCHAVGICYDARRKVTLVQAADIMMGVEPALGPQPVV-VCGIMGPTNTTNNTD
Tgrayi             LRRLLCSNCHAIGICYDARRKVTLVQAADVMMEVEPTLGPQPTV-VCGIVRPPAAAAA--
Tcruzi             LRRFLCSNCHAVGLCYDPRRKFTLVEAADVMMGVEPALGPQPVV-VCGILCPAEPARQD-
                   *  :     :   : ::     ::  .      :   *  : .: :**.

EgracilisZ1        ------------------------------------------------------------
Tborrelli          ------------------------------------------------------------
Pserpens           SSTVPKNIGNGLND-------TRLDLRVSQTAHQRHPDTLPAVSFTTLATASSEASSRSQ
Lbrasiliensis      SVPLDCHKADPTHLSTTM---TALHLSTLPKREGQSLQSLPVDPAVPV--STGALTGELM
Ltarantolae        RAPLNHHKEGSTRLPAVVSSVAALKFSAPSKREDENSDSLPVVSTKPEAEAAGIVSDDWI
Lmexicana          SVPLNRPRVVPARLPAVVSAVTALSLSASPKREAQRSHSVLVGSVESEAGAAGGVADESM
Lmajor             SVPLNYHRAGPARLPAVVSAVTAVGLSAPLVRRDQSSHAVPVGSAESAAEAAGGVAGESM
Linfantum          SAPLHHHRAGPARFPAVVSAATALNLSAPPKKEDECSHSMLVGSAEAE--AAGGVAGEST
Bsaltans           ------------------------------------------------------------
Tvivax             ------------------------------------------------------------
Tcongolense        ------------------------------------------------------------
Tbrucei            ------------------------------------------------------------
Tbgambiense        ------------------------------------------------------------
Tcarassii          A-----------------------------------------------------------
Ttheileri          EIPTTTTNTNTNSNSNS-------------------------------------------
Tgrayi             ------------------------------------------------------------
Tcruzi             ------------------------------------------------------------


EgracilisZ1        ------------------------------------------------------------
Tborrelli          ------------------------------------------------------------
Pserpens           GSTVASATATREATSSPLKSVHPACGAMVWGAGG--------------------------
```

```
Lbrasiliensis      LATPLCGVSNGTKTPLQSSSSSTSTTARLSGVTAESNGDQSDDRLQVNTATSTMALLRAV
Ltarantolae        LAAPLFGATNGCSALHQTKSRSAGIATTLPGGGGEGVDHNNGGPLQADAAISPI------
Lmexicana          LAATMCGVANGCNTSLQASSCPASAAVALHGVVAENCDHESCDRLQADAAVSPV------
Lmajor             LAAPICGVANGCNTSPRASSSPAGTAVTLPGGMAENGAHESCDRLQADAAVSPM------
Linfantum          GAAPMCGAANGCNTPPQASSSPAGTAVRLPGVVAENGGHESCDGLQDDAAVSPI------
Bsaltans           ------------------------------------------------------------
Tvivax             ------------------------------------------------------------
Tcongolense        ------------------------------------------------------------
Tbrucei            ------------------------------------------------------------
Tbgambiense        ------------------------------------------------------------
Tcarassii          ------------------------------------------------------------
Ttheileri          ------------------------------------------------------------
Tgrayi             ------------------------------------------------------------
Tcruzi             ------------------------------------------------------------


EgracilisZ1        --------------------AAHRKVDAHTLAQFPVRPDVRFECSALADRGVGSFFEACV
Tborrelli          -----------------TNNNNNKEVTESDASQITPRCKASIQIYSHNAK---VAFESAI
Pserpens           ------SHSSRSEGLTPPRLSLSIEVSEEDAAGITVRDGGSLHCPIDQMH---VFYDRLL
Lbrasiliensis      PLSSTTPAPALPPFTTATSSSGSLEVSAEDAVGITVRGHSSIQCPLLHPT---PFFEAVV
Ltarantolae        ------LSPRSRPFTMASPHVSALEVSVEDAVGITVRGHSSIHCPLLHPT---PLFEALV
Lmexicana          ------PSPAPPPFTIASPHSSALEVSVNDAVGITVRGHSSIHCPLLHPT---PLFEALV
Lmajor             ------PSPPQPPFAVASPQSRALEVSVEDAVGITVRGHSSIHCPLLHPT---PLFEALV
Linfantum          ------PSPTPPPFTIASPQSSVLEVSVEDAVGITVRGHSSIHCPLLHPT---PLFEALV
Bsaltans           -------------------SQRKREVTVSGAEGITVRCRASIQT--NHAD---EIFEAVL
Tvivax             --------------MVGRHDVPPLEVGESETQDVGEWQSGSLRCPYDRGD---LLFQRIV
Tcongolense        --------------VSRNRGAVEFEVTKEEARAASTRERGSVQCSWAKSE---VLFESVV
Tbrucei            --------------ADAGRGGSAVEVSAEEATEISSRERGSLLCAWHEGK---ILFEHVV
Tbgambiense        --------------ADAGRGGSAVEVSAEEATEISSRERGSLLCAWHEGK---ILFEHVV
Tcarassii          ------HHRSPDALEAPEEGLPVREVKEVHAEGITVRGRGSVQCAWNRSE---RVFQTLV
Ttheileri          ------NTNSTATTMATPIAVSEPAVQEIHAMGITVRGRASLQCQWNNSE---PLFQSLL
Tgrayi             --------------QEIGEKGAPLEVSEVHARDITVRGRGSLQCAWDKGE---MLFQKLV
Tcruzi             --------------LHEAAGTQELEVTEVDASGITVRGRGSLQCAWDNSE---CLFQQLV
                                            *          .    .             ::  :

EgracilisZ1        TACVQKIVQSP------------------------------------------SAAVP--
Tborrelli          QVVLDRLMIGG-------------------------------------------------
Pserpens           CSLIDRLSLAAMANRTSIANLTDAAEE--------------SESNKFSGVSATNSSRT-F
Lbrasiliensis      QSVLDRLVEATVASTSTISEISAVLAMQSGGSGRRNSSTCSAGSLSAGSAPRSTIQAS-S
Ltarantolae        QSVLDVLVEATVAST--------DLTMHGNGNSSQSFPTCHPSSLNGGRASTSAMRVT-S
Lmexicana          QSVLDLLVEATVASTSTISDISAELTMHGSASGAQSFPTSCPSSLSAGSSPASAMRVA-S
Lmajor             QSVLDLLVEATVASTSTISEISAELTTHGSGSGPQSFPTCCPSSLSAGSAPANATRVA-S
Linfantum          QSVLDLLVEATVASTSTISEISAGLTTHGGGSGPQSFPTCCPSSLSAGSAPASAMRVA-S
Bsaltans           QTLLDRLVLAT--------------------------------ATAAAASPASKLGGA--
Tvivax             QCLLDRIALAT--------------------------------SVVSFNSPSNSSCIP-N
Tcongolense        QCLLDRIALGT--------------------------------SALPFSPSVRSSGEY-V
Tbrucei            QCLLDCIALGT--------------------------------SAISFTAPVSPLGVP-V
Tbgambiense        QCLLDCIALGT--------------------------------SAISFTAPVSPLGVP-V
Tcarassii          QCLLDRLSLAT--------------------------------STAAGVRVST------E
Ttheileri          QSLLDRLAMAT--------------------------------ATSSVVAAAASNGRAKI
Tgrayi             QCLLDRLALAT--------------------------------TTSSVIATTTPTGRVRR
Tcruzi             QSLLDRLAMAT--------------------------------ATSSVVSAASTALLA-G
                      :: :  .

EgracilisZ1        ---------------------PPPNSTPVD--
Tborrelli          ---------RQSHELISLTTNPSVFDLLID--
Pserpens           AKRDPKVDKVIAEELLKLTMQPSTLNILLDR-
Lbrasiliensis      VRRRPHASRAIVQDLLNLTMQPCALDILLDRK
Ltarantolae        ARRRPHASRAIMEDLLNLTMQPCSLDILLDRK
Lmexicana          TRRRPKASRAIMEDLLNLTMQPCALDILLDRK
Lmajor             TRRRPHASRAIMEDLRNLTMQPCALDILLDRK
Linfantum          TRRRPHASRAIMEDLLNLTMQPCALDILLDRK
Bsaltans           -----AAARTAAQELLEISSNPSVMDVLLDGK
Tvivax             EGAFSVTNHTAAHELLKISTTPSPIDVLIN--
Tcongolense        THEEPLANRTVAQELLQITVCPSAIDILLN--
Tbrucei            DSEEFSVNRSTAQELLRITMCPSAIDVLLD--
Tbgambiense        DSEEFSVNRSTAQELLRITMCPSAIDVLLD--
Tcarassii          DEKERVANRTAALELLQLTLNPSAIDVLLD--
```

```
Ttheileri          DEKESATNLAVAQELLRITLNPSALDILLE--
Tgrayi             DERETSMNLNVAQELLQITMQPSPLDVLL---
Tcruzi             DEKEATVNCNIAQELLRITLDPSPLDVLMDSN
                                        *   .  :
```

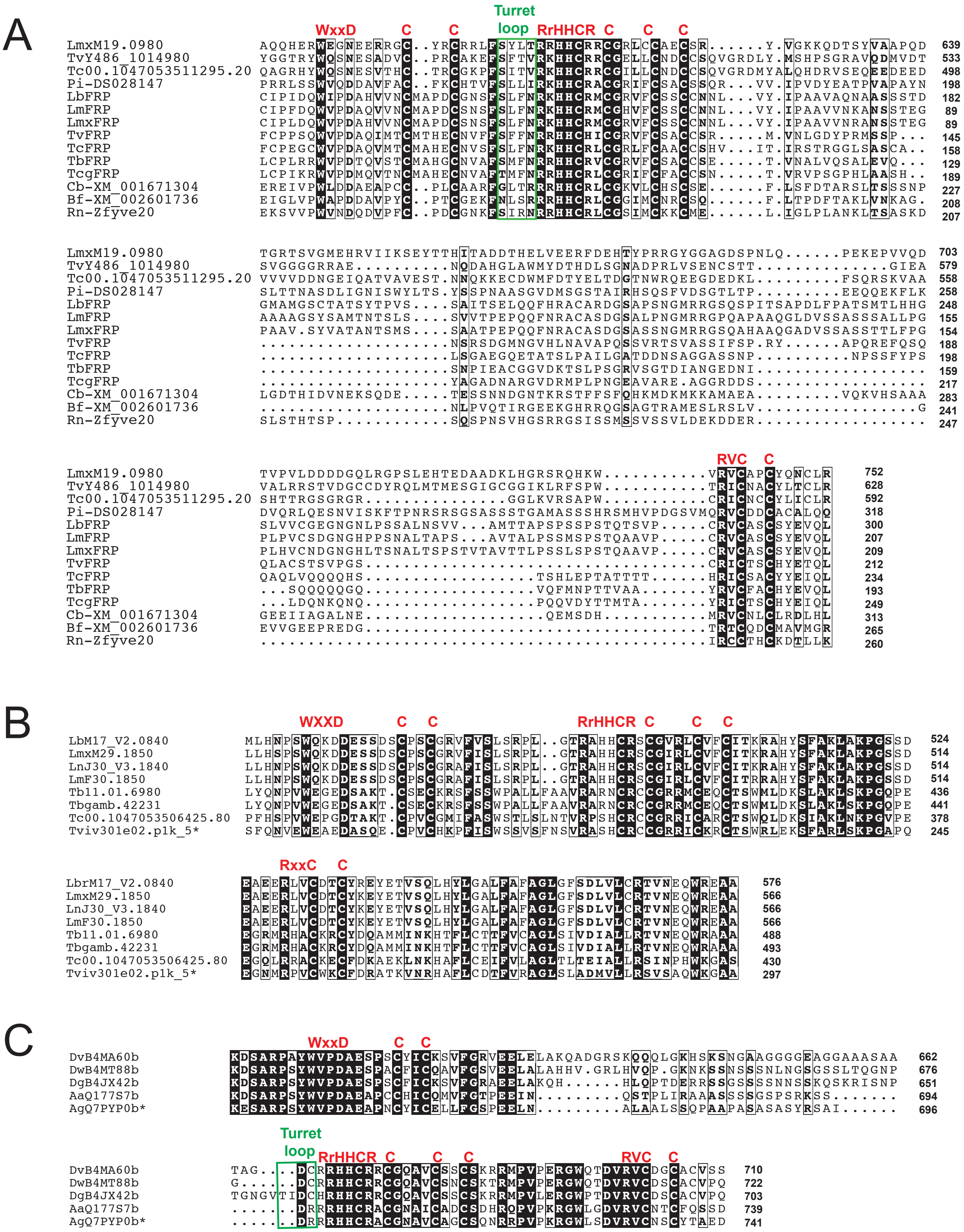
A
WxxD
C
C
Turret loop
RrHHCR
C
C
C
RVC
C
LmxM19.0980
TvY486_1014980
Tc00.1047053511295.20
Pi-DS028147
LbFRP
LmFRP
LmxFRP
TvFRP
TcFRP
TbFRP
TcgFRP
Cb-XM_001671304
Bf-XM_002601736
Rn-Zfyve20
B
WXXD
C
C
RrHHCR
C
C
C
RxxC
C
LbM17_V2.0840
LmxM29.1850
LnJ30_V3.1840
LmF30.1850
Tb11.01.6980
Tbgamb.42231
Tc00.1047053506425.80
Tviv301e02.p1k_5*
LbrM17_V2.0840
C
WxxD
C
C
Turret loop
RrHHCR
C
C
C
RVC
C
DvB4MA60b
DwB4MT88b
DgB4JX42b
AaQ177S7b
AgQ7PYP0b*

Table S1: Synthetic oligonucleotides used in this study. All sequences are written with the 5' end left-most, and where relevant restriction endonuclease sites are underlined.

**Designation**	**Sequence**	**Purpose**
qRT For:	GCCAAACCGACAATTTCACT	qRT-PCR
qRT Rev:	CTCCCGCATTAATGACGTTT	qRT-PCR
β-tubulin For:	CAAGATGGCTGTCACCTTCA	qRT-PCR
β-tubulin Rev:	GCCAGTGTACCAGTGCAAGA	qRT-PCR
TbFRP-RNAi-A For:	GAGCGGGAGATGGGTAATGG	qRT-PCR
TbFRP-RNAi-A Rev:	TGAACTCCGAGTTTCGTCTC	qRT-PCR
TbFRP-RNAi-B For:	GAAGAGCGGGAGATGGGTAAT	qRT-PCR
TbFRP-RNAi-B Rev:	TGTGGGGTTCATAAACTGGA	qRT-PCR
TbFRP For:	GCCAAAGCTTATGGATCATATTGATCTTGC	Ectopic expression of TbFRP
TbFRP Rev:	GCTAGGGCCCGTCCAATAGCACGTCAATAGC	Ectopic expression of TbFRP
TbFRP-FYVE Rev:	GCGGGCCCCACAACAAGCTGTACTTCATAG	Ectopic expression of FYVE domain
TbFRP-FYVE-Fbox Rev:	GCGGGCCC GGCAGCACCTGCGGATG	Ectopic expression of FYVE domain
TbFRP Tag For:	GTTCTCCGTCAACCGCTCAACAGCGCAGGAGCTATTGCGAATT<br>ACGATGTGCCCATCGGCTATTGACGTGCTATTGGACGGTACCG<br>GGCCCCCCCTCGAG	Genomic tagging of TbFRP
TbFRP Tag Rev:	CACAAACCCAGCTGGCAAAATAGTAAAACGGTACACTTGCCA<br>CACAACAAATTACCTCATTCCAACCATGTATGGCGGCCGCTCT<br>AGAACTAGTGGAT	Genomic tagging of TbFRP

Table S2: Number of amino acid residues between RrHHCR + 10 and RVC motifs in FYVE Domains

Species \ Gene ID	Tb09.160.3480	Tb09.211.1970	Tb02.211.2990	Tb10.05.0240	Tb927.7.1840	Tb927.7.1880	Tb11.01.6980	Tb11.01.7720
T. brucei	16	16	16	13	16	16	23	21
T. b. gambiense	16	16	16	13	16	16	23	21
T. cruzi	00	16	16	14	16	16	23	21
T. vivax	16	-	16	14	16	16	23	-
T. congolense	16	16	16	13	16	16	-	21
L. major	16	16	-	14	-	-	23	-
L. mexicana	16	16	16	14	-	-	23	-
L. braziliensis	16	16	16	14	-	-	23	-
L. infantum	16	16	-	14	-	-	23	-

Species \ Gene ID	Tb10.61.0730	Tb11.47.0002	Tb927.7.690	Tb927.7.3790	Tb937.7.530
T. brucei	118	29	59	68	17
T. b. gambiense	-	29	63	68	17
T. cruzi	107	29	65	83	17
T. vivax	107	29	78	71	17
T. congolense	107	29	-	68	17
L. major	119	70	-	127	36
L. mexicana	119	70	-	129	36
L. braziliensis	119	71	-	127	36
L. infantum	118	70	-	-	36

Protein \ Species	Rn	Bt	Hs	Mm	Am	Cb	Ce	Dm	Bf	Pi
Rabenosyn-5	59	59	59	59	59	92	98	32	62	-
PIK-D	-	-	-	-	-	-	-	-	-	129

Key: Rn (*Rattus norvegicus*), Bt (*Bos taurus),* Hs (*Homo sapiens*), Mm (*Mus musculus*), Am (*Ailuropoda melanoleuca*), Cb (*Caenorhabditis briggsae*), Ce (*Caenorhabditis elegance*), Dm (*Drosophila melanogaster*) Bf (*Branchiostoma floridae*), Rn (*Rattus norvegicus*) and Pi (*Pichia pastoris*).
